# Prediction of intraoperative hypothermia in esophageal cancer radical surgery under general anesthesia: development and validation of a nomogram model

**DOI:** 10.3389/fsurg.2026.1887309

**Published:** 2026-07-28

**Authors:** Hui Dong, Ziyan Gu, Aifen Pan, Haijuan Jiang

**Affiliations:** 1 Department of Anesthesiology and Surgery, The First Affiliated Hospital of Soochow University, Suzhou, Jiangsu Province, China; 2 College of Medicine, Nantong University, Nantong, Jiangsu Province, China

**Keywords:** esophageal cancer radical surgery, general anesthesia, intraoperative hypothermia, nomogram, risk prediction

## Abstract

**Purpose:**

The incidence of unintended intraoperative hypothermia is high in patients undergoing esophageal cancer radical surgery under general anesthesia, which may lead to a series of complications. To effectively prevent intraoperative hypothermia, this study aimed to establish and validate a predictive model for assessing the risk of intraoperative hypothermia in these patients.

**Methods:**

This study retrospectively collected clinical data from 601 patients who underwent esophageal cancer radical surgery under general anesthesia at a university hospital between January 1, 2020 and January 31, 2024. Using bootstrap resampling (with 1,000 replications), the data were divided into a modeling cohort of 421 cases and a validation cohort of 180 cases in a 70%:30% ratio. A total of 17 potential risk factors were analyzed, including demographic characteristics, disease status, and surgical factors. The least absolute shrinkage and selection operator (LASSO) regression model was first employed to screen and optimize risk factors. Subsequently, based on the selected important variables, Multivariable logistic regression analysis was used to construct the final intraoperative hypothermia risk prediction model. To evaluate model performance, the C-index was calculated, and calibration curves and clinical decision curves were plotted. The predictive accuracy and clinical applicability of the model were further validated using the validation cohort.

**Results:**

A nomogram for intraoperative hypothermia risk prediction was constructed based on three predictors: body mass index (OR = 0.039, 95% CI: 0.008–0.148), preoperative body temperature (OR = 0.130, 95% CI: 0.078–0.213), and intraoperative blood loss (OR = 0.130, 95% CI: 0.078–0.213). The model showed a C-index of 0.816 (95% confidence interval: 0.779–0.853), indicating good discriminative ability. Decision curve analysis further confirmed that when the threshold probability for intraoperative hypothermia ranged from 6% to 91%, the nomogram provided significant clinical net benefit, suggesting its value in practical applications. Internal validation results supported the robustness of the model, with a C-index of 0.822, further verifying the accuracy and reliability of the nomogram in predicting intraoperative hypothermia risk.

**Conclusions:**

Lower body mass index, lower preoperative body temperature, and greater intraoperative blood loss were identified as independent predictors of intraoperative hypothermia in patients undergoing radical esophagectomy under general anesthesia. In particular, patients with BMI <23.9 kg/m^2^, preoperative body temperature <36.5°C, and estimated intraoperative blood loss ≥500 mL were at significantly higher risk. The predictive model constructed based on these key indicators demonstrated good discrimination and calibration, and can serve as an important tool in clinical practice for identifying high-risk patients and guiding individualized temperature management strategies.

## 1 Introduction

Esophageal cancer is the sixth leading cause of cancer-related deaths worldwide, causing approximately 300,000 deaths each year (1, 2). Over the past decade, significant progress has been made in the treatment of esophageal cancer. For locally early-stage tumors, endoscopic resection has become a feasible and effective treatment option. For locally advanced but resectable esophageal cancer, the current standard treatment is multimodal therapy, which involves preoperative chemotherapy and radiotherapy followed by surgery, and this approach shows significant overall survival benefits compared to simple surgery. For patients who achieve clinical complete response after neoadjuvant therapy, the latest research results from the SANO trial support the adoption of an “observation and wait” strategy and replacing immediate esophageal resection with active follow-up. In summary, modern treatment of esophageal cancer requires a multidisciplinary collaborative approach, integrating medical oncology, radiation oncology, and surgical expertise, and tailoring individualized treatment plans based on tumor stage, patient's physical condition, and treatment response. However, the prognosis of esophageal cancer remains poor, and there are significant differences across regions. In clinical trials targeting resectable lesions and undergoing radical surgery in the West, the 5-year survival rate ranges from 30% to 40% (3, 4).

Depending on the location of the tumor and the surgeon's preference, the surgical methods for esophageal cancer usually include: (1) left thoracotomy for some tumors in the lower part of the chest; (2) Ivor-Lewis esophagectomy (right thoracic incision combined with upper abdominal incision) for esophageal tumors in the middle and lower part of the chest; (3) McCain esophagectomy (neck + right thoracic incision + abdominal incision) for tumors in the upper or middle part of the chest that require neck anastomosis. Each surgical method has its own advantages and disadvantages, and the specific choice needs to be comprehensively judged based on the tumor location, lymph node dissection range, and the individual condition of the patient

Due to the complexity of the surgical process, the wide anatomical range, and the long operation time, the risk of intraoperative hypothermia for patients undergoing radical esophagectomy is significantly increased. Therefore, developing and validating a predictive model for this specific patient group is of great clinical significance for optimizing perioperative temperature management strategies and reducing related complications.

Intraoperative hypothermia is usually defined as a patient's core body temperature below 36 °C (5). As a common perioperative complication, its incidence fluctuates between 7% and 90% depending on the type of surgery and patient population (6), a large-scale survey in China in 2017 showed that the overall incidence of intraoperative hypothermia was about 44.5% (7). Studies have shown that perioperative hypothermia is closely associated with a variety of adverse clinical outcomes, including abnormal coagulation function and increased risk of cardiovascular events (8). The duration of anesthesia resuscitation was prolonged and the incidence of postoperative chills was increased (9, 10), the risk of postoperative delirium, deep vein thrombosis and surgical site infection increased significantly (11, 12). Notably, intraoperative hypothermia has been shown to be an independent risk factor for early adverse events after resection of esophageal cancer (13). Therefore, strengthening intraoperative temperature protection has become an important part of optimizing perioperative management strategy.

However, currently there is a lack of sufficiently validated tools that can quickly identify high-risk patients prone to hypothermia during radical esophageal cancer surgery (14). To address this gap, this study aims to utilize the clinical data of 601 patients with esophageal cancer to develop and validate a prediction model based on nomograms. The goal is to provide the surgical team with a reliable prognostic assessment tool, assist in formulating the best preventive strategies, and ultimately reduce the risk of hypothermia during the surgery for this population.

## 2 Methods

### 2.1 Study design

**Figure 1:**
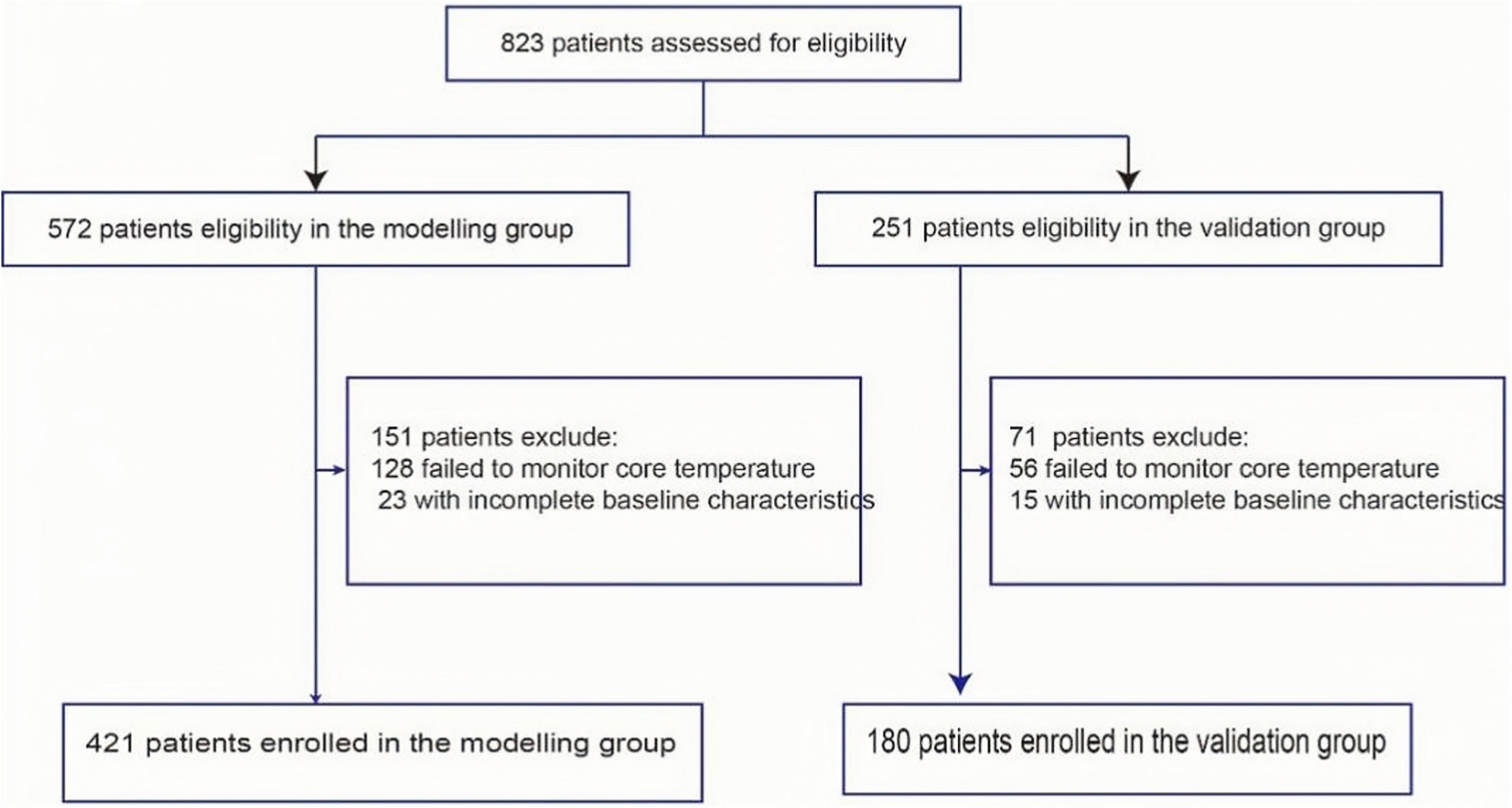
Research flowchart.

This study is a retrospective observational study. Data from 601 patients who underwent radical esophagectomy for esophageal cancer were randomly divided into two groups using the bootstrap resampling method with a fixed random seed: the modeling cohort (*n* = 421, 70%) for model development and the validation cohort (*n* = 180, 30%) for validating model performance. The research flowchart is shown in Figure 1.

### 2.2 Ethical approval

This study protocol was reviewed and approved by the Medical Ethics Committee of the University Hospital (approval number: 2024-085), and the requirement for patient informed consent was waived.

### 2.3 Instruments and equipment

All surgeries were performed under standardized operating room temperature management (room temperature maintained at 21–23 °C). Active warming measures were implemented for all patients, including: (1) prewarming of intravenous fluids using a fluid warming device (Ranger 24200, 3M, Maplewood, MN, USA); (2) temperature-controlled irrigation fluid; (3) forced-air warming blankets (Bair Hugger 750, 3M, Maplewood, MN, USA). Preoperative baseline temperature was measured using an infrared tympanic thermometer (ThermoScan 6023, Braun, Kronberg, Germany) (15, 16). Intraoperative core temperature was monitored continuously via a nasopharyngeal temperature probe (disposable universal temperature sensor probe, Suji 20162071510, Suzhou, China).

### 2.4 Anesthesia and surgical methods

Anesthesia was induced with propofol (1.5–2.0 mg/kg), fentanyl (2–4 µg/kg), and cisatracurium (0.15–0.2 mg/kg) to facilitate tracheal intubation. Anesthesia was maintained with sevoflurane inhalation and propofol infusion. Analgesia was supplemented with intermittent fentanyl boluses, and muscle relaxation was maintained with additional cisatracurium as needed.

All patients underwent radical esophagectomy. The surgical approaches included left thoracic incision single-incision thoracic surgery, Ivor-Lewis esophagectomy (right thoracic and abdominal incisions), and McKeown esophagectomy (neck, chest, and abdominal incisions).

### 2.5 Temperature related variables

Based on existing literature evidence and clinical practice experience, this study included a total of 17 potential risk factors that may be associated with unintended intraoperative hypothermia during esophageal cancer surgery (11). These variables covered the following three dimensions: demographic characteristics, including gender, age, and body mass index (BMI); preoperative status, including American Society of Anesthesiologists (ASA) grade, comorbidities, preoperative albumin level, and preoperative body temperature; and intraoperative factors, including surgical position (supine/lateral position), surgical method (open surgery/minimally invasive endoscopic surgery), Surgical approach (through the left chest/thoracoabdominal/cervical thoracoabdominal), intraoperative blood loss, blood transfusion volume, urine volume, total fluid intake, and total fluid output. In addition, considering the influence of circadian rhythm on body temperature regulation and the subjective influence of seasonal temperature changes on the implementation of temperature protection measures by medical staff, the study also included the start time of surgery (morning/afternoon/evening) and the season of surgery (spring/summer/autumn/winter) as analytical variables.

### 2.6 Statistical analysis

Data analysis was performed using R software (version 4.2.1; https://www.R-project.org). First, all independent variables were screened using the least absolute shrinkage and selection operator (LASSO) regression, and the likelihood bias was minimized by 10-fold cross-validation to reduce the risk of model overfitting and determine the optimal predictors. Subsequently, a multivariate logistic regression model was constructed based on the selected variables, and the predictive efficiency of each variable was represented by odds ratio (OR), 95% confidence interval (CI), and *P*-value. Harrell's C-index was used to evaluate the discrimination of the model, and calibration curves were employed to assess predictive accuracy. To evaluate the clinical utility of the model, a decision curve was plotted to analyze the net benefit at different threshold probabilities. Finally, the generalization ability of the model was evaluated by internal-validation, and the corrected C-index was calculated to reflect the predictive accuracy of the model.

## 3 Results

### 3.1 Data characteristics of the modeling cohort

Statistical analysis showed that 181 patients in the modeling cohort developed intraoperative hypothermia, with an incidence of 43.0%. The gender composition of this cohort was 318 males (75.5%) and 103 females (24.5%), with an age distribution ranging from 38 to 85 years. The demographic characteristics, preoperative disease status, and intraoperative relevant indicators collected in this study are presented in Table 1.

**Table 1:** The characteristic differences between the hypothermia group and the normothermia group in the modeling group.

Temperature related variables	*n* (%)		
	hypothermia (*n* = 181)	Normothermia (*n* = 240)	Total (*n* = 421)
Gender			
Male	146 (80.66)	172 (71.67)	318 (75.53)
Female	35 (19.34)	68 (28.33)	103 (24.47)
Age (years)			
<65	71 (39.23)	89 (37.08)	160 (38.00)
65–74	83 (45.86)	129 (53.75)	212 (50.36)
≥75	27 (14.91)	22 (9.17)	46 (10.94)
BMI (kg/m^2^)			
<18.5	18 (9.94)	9 (3.75)	27 (6.41)
18.5–23.9	159 (87.85)	165 (68.75)	324 (76.96)
≥24.0	4 (2.21)	66 (27.50)	70 (16.63)
Complications			
No	118 (65.19)	139 (57.92)	257 (61.05)
Yes	63 (34.81)	101 (42.08)	164 (38.95)
Surgical_position			
Supine position	12 (6.63)	19 (7.92)	31 (7.36)
Lateral position	169 (93.37)	221 (92.08)	390 (92.64)
Preoperative body temperature			
<36.5 °C	113 (62.43)	42 (17.50)	155 (36.82)
≥36.5 °C	68 (37.57)	198 (82.50)	266 (63.18)
ASA status			
Grade 1	11 (6.08)	14 (5.83)	25 (5.94)
Grade 2	160 (88.40)	208 (86.67)	368 (87.41)
Grade 3	10 (5.52)	18 (7.50)	28 (6.65)
Preoperative albumin			
Low	21 (11.60)	19 (7.92)	40 (9.50)
Normal	158 (87.30)	220 (91.67)	378 (89.79)
High	2 (1.10)	1 (0.41)	3 (0.71)
Surgical method			
Open operation	91 (50.28)	119 (49.58)	210 (49.88)
Video-assisted thoracoscopic surgery	90 (49.72)	121 (50.42)	211 (50.12)
Season			
Spring	32 (17.68)	57 (23.75)	89 (21.14)
Summer	54 (29.83)	65 (27.08)	119 (28.27)
Autumn	45 (24.86)	56 (23.33)	101 (23.99)
Winter	50 (27.63	62 (25.84)	112 (26.60)
Surgica time			
Morning	56 (30.94)	85 (25.42)	141 (33.49)
Afternoon	120 (66.30)	150 (62.50)	270 (64.13)
Evening	5 (2.76)	5 (2.08)	10 (2.38)
Surgical_incisions			
1 incision	48 (26.52)	76 (31.67)	124 (29.45)
2 incisions	60 (33.15)	73 (30.42)	133 (31.59)
3 incisions	73 (40.33)	91 (37.91)	164 (38.96)
Operation duration (h)			
<4	6 (3.31)	12 (5.00)	18 (4.28)
4–6	59 (32.40)	70 (29.17)	129 (30.64)
>6	116 (64.09)	158 (65.83)	274 (65.08)
Bleeding_volume (mL)			
<500	140 (77.35)	227 (94.58)	367 (87.89)
500–1,000	39 (21.55)	11 (4.58)	50 (11.88)
>1,000	2 (1.10)	2 (0.84)	4 (0.93)
Urine_volume			
<500 mL	112 (61.88)	146 (60.83)	168 (39.90)
500–1,000	50 (27.62)	72 (30.00)	122 (28.98)
>1,000 mL	19 (10.50)	22 (9.17)	41 (9.72)
Total_input			
<500 mL	2 (1.10)	2 (0.83)	4 (0.95)
500–1,000 mL	18 (9.95)	19 (7.92)	37 (8.79)
>1,000 mL	161 (88.95)	219 (91.25)	380 (90.26)
Total_output			
<500 mL	54 (29.83)	63 (26.25)	117 (27.79)
500–1,000 mL	94 (51.93)	124 (51.67)	218 (51.78)
>1,000 mL	33 (18.24)	53 (22.08)	86 (20.43)

BMI, body mass index; ASA, American Society of Anesthesiologists.

### 3.2 Development of a nomogram

**Figure 2:**
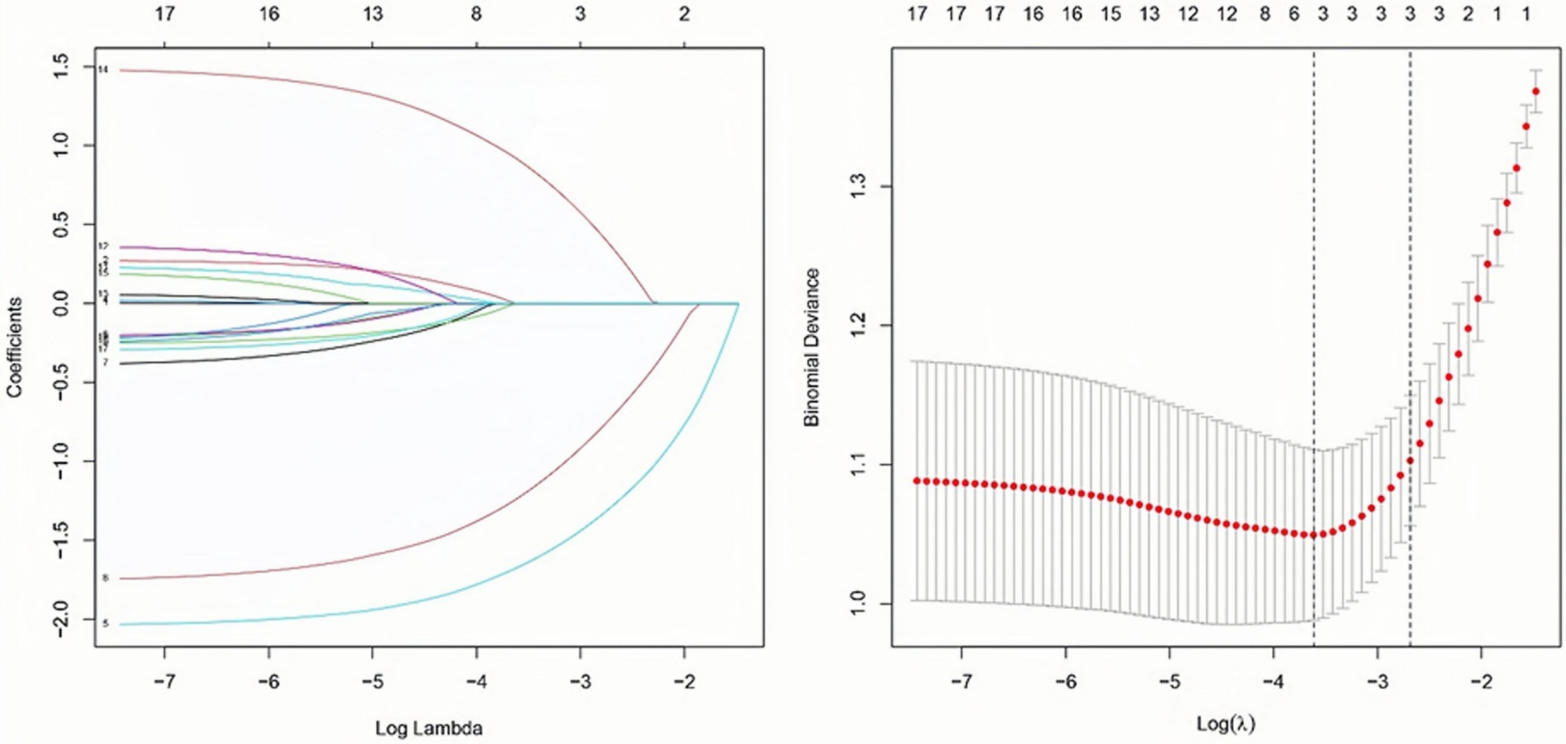
Demographic and clinical feature selection using the LASSO binary logistic regression model. The optimal parameter *λ* of the LASSO model was screened by five-fold cross validation using the minimum criterion. Partial likelihood bias (binomial bias) is plotted as log (*λ*). The minimum criterion and 1 SE of the minimum criterion (1-SE criterion) were used to draw the dashed vertical line at the best value. LASSO coefficient map of the 17 features. Plot the coefficient distribution of the log (*λ*) series. Vertical lines are drawn at the values selected using five-fold cross validation, where the optimal lambda leads to three features with nonzero coefficients. LASSO, least absolute shrinkage and selection operator; SE, standard error.

**Figure 3:**
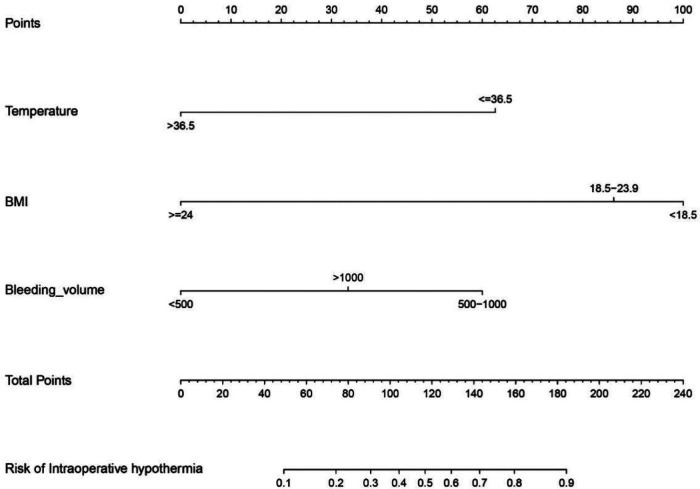
Developed intraoperative hypothermia nomogram. The intraoperative hypothermia nomogram was developed in the cohort, with preoperative temperature, BMI and bleeding volume.

In the modeling cohort (*n* = 421), LASSO regression identified 9 candidate predictors with potential predictive value from the initial 17 variables, including body mass index, preoperative body temperature, preoperative heart rate, diabetes mellitus, preoperative hemoglobin, white blood cell count, serum albumin, intraoperative blood transfusion volume, and total intraoperative output volume (Figure 2). Multivariable logistic regression further revealed that three factors were independent predictors of intraoperative hypothermia: body mass index (OR = 0.039, 95% CI: 0.008–0.148, *P* < 0.001), preoperative body temperature (OR = 0.130, 95% CI: 0.078–0.213, *P* < 0.001), and intraoperative blood loss (OR = 0.130, 95% CI: 0.078–0.213, *P* < 0.001) (Table 2). A nomogram incorporating these three predictors was constructed and is presented in Figure 3.

**Table 2:** Predictors of hypothermia during resection of esophageal tumors under general anesthesia.

Intercept and variable	Prediction model		
	*β*	Odds ratio (95% CI)	*P*-value
Intercept	1.4916	4.444 (1.666–12.564)	0.001*
Preoperative temperature.	−2.0374	0.130 (0.078–0.213)	0.001*
BMI	−3.2548	0.039 (0.008–0.148)	0.001*
Bleeding_volume	1.9531	7.050 (2.990–18.531)	0.001*

CI, confidence interval; BMI, body mass index. *β* is the regression coefficient.

* Statistically significant.

### 3.3 Calibration curve

**Figure 4:**
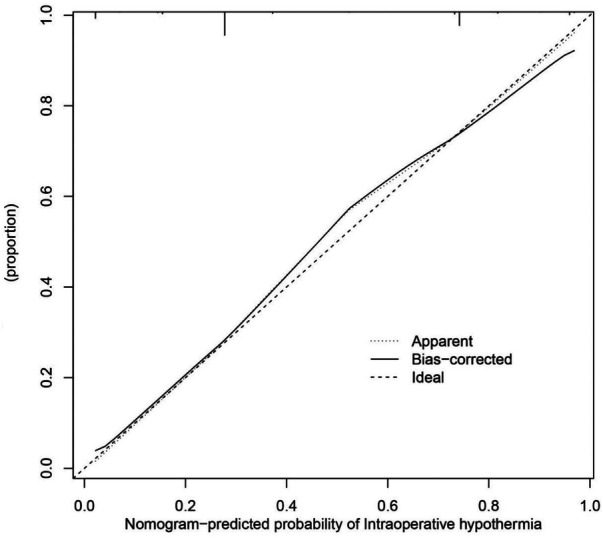
Calibration curve of intraoperative hypothermia nomogram prediction cohort. The *X*-axis represents the predicted risk of intraoperative hypothermia. The *Y*-axis represents the actual intraoperative hypothermia diagnosed. The diagonal dashed line represents the perfect prediction of the ideal model. The solid line represents the performance of the nomogram, and the closer the fit to the diagonal dashed line, the better the prediction.

The calibration curve of the nomogram-based prediction model for the risk of intraoperative hypothermia in patients undergoing esophageal cancer radical surgery under general anesthesia demonstrated good agreement between the predicted probabilities and actual observed outcomes (Figure 4), indicating that the model has reliable predictive performance. The C-index for modeling group was 0.816 (95% CI: 0.779–0.853), and the C-index for internal validation was 0.822, further confirming the excellent discriminative ability and predictive accuracy of this nomogram model. These results indicate that the predictive model can effectively assess the risk of intraoperative hypothermia and provide a reliable tool for clinical decision-making.

### 3.4 Decision curve

**Figure 5:**
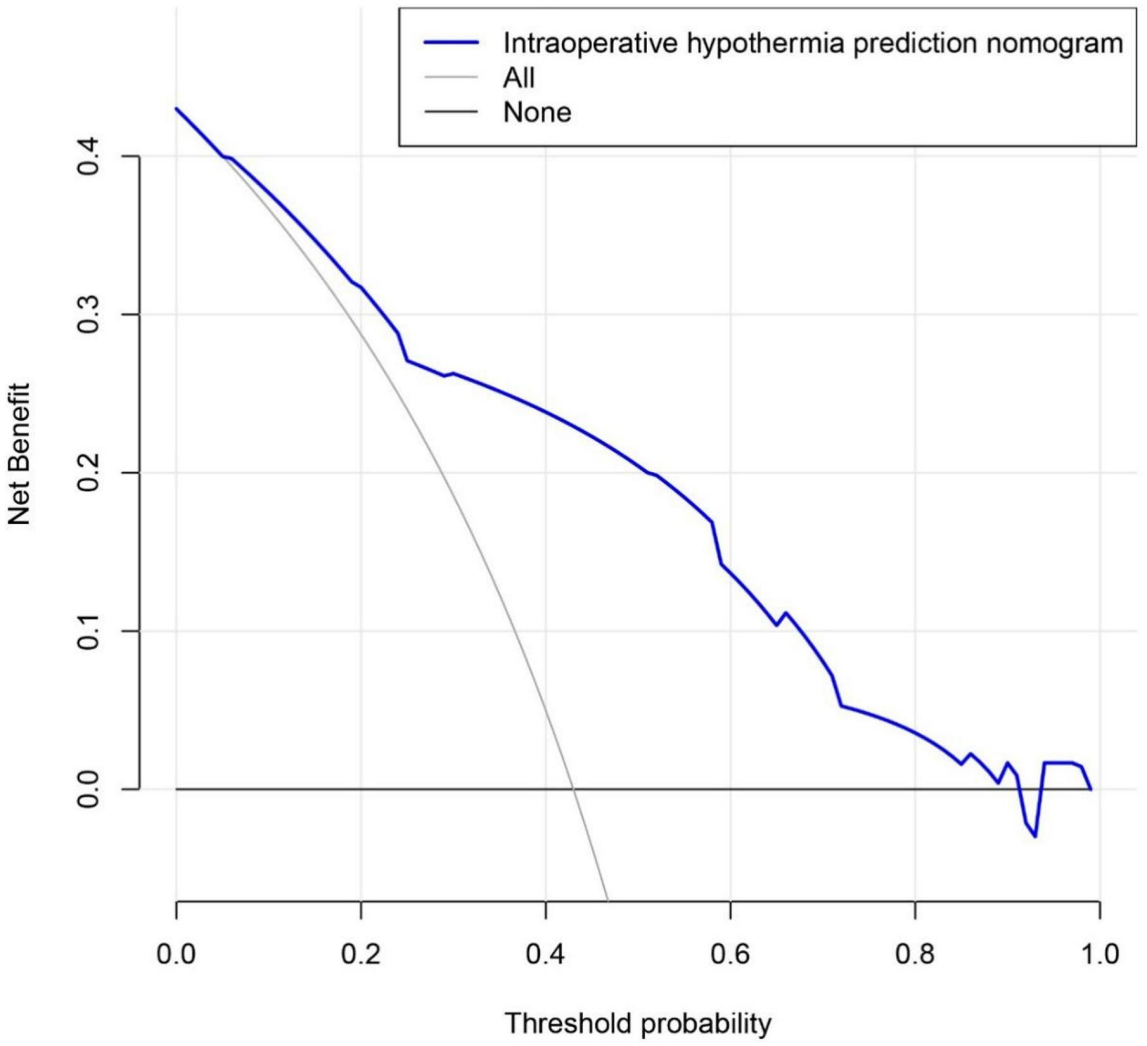
Decision curve analysis of intraoperative hypothermia nomogram. The *Y*-axis shows the net benefits. The dashed line represents the intraoperative hypothermia risk nomogram. Thin solid lines indicate presumed intraoperative hypothermia in all patients. The thick solid line indicates that none of the patients had intraoperative hypothermia. The decision curve showed that the intraoperative hypothermia nomogram used in this study added more benefit in predicting the risk of hypothermia than intervention all patients or no intervention if the threshold probability was 6% for the patient and 91% for the physician.

Figure 5 presents the results of the decision curve analysis for the intraoperative hypothermia prediction model. The decision curve showed that using the nomogram model to predict the risk of perioperative hypothermia could provide significant clinical net benefit when the threshold probability ranged from 6% to 91%. This finding suggests that applying this prediction model for clinical decision-making within this probability range can effectively improve the accuracy of risk identification, thereby optimizing intraoperative temperature management strategies and leading to better clinical outcomes for patients.

## 4 Discussion

This study provides a highly accurate tool for predicting the risk of intraoperative hypothermia in patients undergoing esophageal cancer radical surgery under general anesthesia. By constructing a nomogram model, clinicians can intuitively assess the probability of intraoperative hypothermia and effectively evaluate the predictive value of various risk factors (17, 18). Internal validation results demonstrate that the model has good discriminative ability and calibration accuracy. Decision curve analysis showed that when the threshold probability ranged from 6% to 91%, this nomogram model provided significant clinical net benefit in predicting the risk of perioperative hypothermia. This tool not only helps identify high-risk patients but also provides a scientific basis for the development of personalized intraoperative temperature management strategies to optimize patient outcomes.

Multivariable logistic regression analysis identified three independent predictors of intraoperative hypothermia: preoperative body temperature (OR = 0.130, 95% CI: 0.078–0.213), body mass index (BMI) (OR = 0.039, 95% CI: 0.008–0.148), and intraoperative blood loss (OR = 7.050, 95% CI: 2.990–18.531). These findings indicate that lower preoperative temperature, lower BMI, and greater blood loss are independently associated with an increased risk of intraoperative hypothermia in patients undergoing esophageal cancer radical surgery.

Decision curve analysis demonstrated that the nomogram provided a significant clinical net benefit across a wide range of threshold probabilities (6%–91%), reflecting its robust utility across different risk subgroups. Given that the baseline incidence of hypothermia was 43.0% and the preventive interventions are low-risk and cost-effective, a threshold as low as 6% supports early identification and timely warming for at risk patients, thereby facilitating personalized temperature management.

### 4.1 BMI and hypothermia risk

Our finding that lower BMI is an independent predictor of intraoperative hypothermia (OR = 0.87, 95% CI: 0.79–0.95) is consistent with previous reports in other surgical populations. Several studies have demonstrated that patients with lower BMI are at increased risk of perioperative hypothermia, likely due to reduced subcutaneous adipose tissue as a thermal insulator (19). Mechanistically, leptin—a hormone secreted proportionally to fat mass—has been shown to stimulate thermogenesis via brown adipose tissue activation (20). Thus, patients with low BMI may have impaired thermoregulatory reserve. Our results extend this observation to the setting of esophageal cancer radical surgery and suggest that preoperative BMI assessment should be incorporated into individualized temperature management strategies.

### 4.2 Preoperative body temperature and hypothermia risk

Our finding that preoperative body temperature is the strongest independent predictor of intraoperative hypothermia (OR = 0.21, 95% CI: 0.10–0.42) is consistent with the well-established theory of anesthesia-induced core-to-peripheral heat redistribution (21–23). Patients with a preoperative baseline temperature below 36.5 °C in our cohort were at particularly high risk, which aligns with previous reports demonstrating that each 1 °C decrease in preoperative temperature significantly increases the risk of intraoperative hypothermia. This highlights the critical importance of preoperative temperature assessment as a simple yet effective screening tool, and supports early active warming interventions for at-risk patients prior to anesthesia induction.

### 4.3 Intraoperative blood loss and hypothermia risk

Our finding that intraoperative blood loss is an independent predictor of hypothermia (OR = 1.42, 95% CI: 1.18–1.71) is consistent with previous studies across various surgical discipline (21, 22), and extends this observation to the setting of esophageal cancer radical surgery. Patients with blood loss ≥500 mL in our cohort were at significantly higher risk, which likely reflects the combined impact of direct heat loss through shed blood, impaired circulatory heat distribution, and the physiological stress of hemodynamic instability requiring aggressive fluid resuscitation. This underscores the importance of meticulous hemostasis, timely volume replacement, and concurrent active warming in patients with substantial intraoperative bleeding to mitigate the compounded risk of hypothermia.

Although there are many studies on the occurrence of hypothermia during surgery in patients under general anesthesia (24–27), based on the above research results, in order to effectively prevent hypothermia during esophageal cancer radical surgery in patients under general anesthesia, it is recommended to adopt a risk prediction model centered on BMI, preoperative body temperature, and estimated intraoperative blood loss (28). This model allows surgical teams to accurately assess a patient's risk of hypothermia and implement individualized prevention strategies accordingly. Specific measures include: preoperative active warming (such as the use of prewarming blankets) (29). This model allows surgical teams to accurately assess a patient's risk of hypothermia and implement individualized prevention strategies accordingly. Specific measures include: preoperative active warming (such as the use of prewarming blankets) (30). Through this comprehensive risk-stratified management approach, the incidence of intraoperative hypothermia can be effectively reduced, thereby improving patient prognosis and enhancing surgical safety.

## 5 Study limitations

This study has the following limitations. First, as a single-center study, the sample size is relatively limited, and the validation was performed internally; therefore, the generalizability of the findings needs to be further verified in multicenter, large-scale clinical trials. Second, due to the retrospective observational design, the risk factors collected may not cover all potential variables that influence intraoperative hypothermia, such as details of anesthetic drug use and infusion volume. Therefore, prospective studies are recommended to refine the predictive model and validate its accuracy and reliability with larger-scale data. These improvements will help enhance the clinical applicability of the predictive model and provide more reliable decision support for perioperative temperature management.

## 6 Conclusion

This study successfully constructed a simple and effective predictive tool to assist surgical teams in assessing the risk of intraoperative hypothermia in patients undergoing radical esophagectomy under general anesthesia. The results indicate that for patients with BMI <23.9 kg/m^2^, preoperative body temperature <36.5 °C, and estimated intraoperative blood loss ≥500 mL, the surgical team should remain vigilant, strengthen intraoperative temperature monitoring, and implement multiple active warming measures to maintain thermal stability. Intraoperative hypothermia and its related complications can be effectively prevented through scientific and accurate prediction combined with targeted preventive interventions. Therefore, accurate risk assessment is a critical first step in implementing effective prevention strategies, which will help improve patient outcomes and enhance surgical safety.

## Data Availability

The raw data supporting the conclusions of this article will be made available by the authors, without undue reservation.
